# Comparative study on 3D morphologies of delignified, single tracheids and fibers of five wood species

**DOI:** 10.3762/bjnano.17.16

**Published:** 2026-02-04

**Authors:** Helen Gorges, Felicitas von Usslar, Cordt Zollfrank, Silja Flenner, Imke Greving, Martin Müller, Clemens F Schaber, Chuchu Li, Stanislav N Gorb

**Affiliations:** 1 Department of Functional Morphology and Biomechanics, Zoological Institute, Kiel University, Kiel, Germany; 2 TUM Campus for Biotechnology and Sustainability, Technical University of Munich, Straubing, Germany; 3 Helmholtz-Zentrum Hereon, Geesthacht, Germany

**Keywords:** 3D models, delignification, tracheid, wood, X-ray nanotomography

## Abstract

Wood tracheids and fibers exhibit diverse structures and shapes across plant species. The hierarchical structure and composition of cellulose, hemicelluloses, and lignin enables wood to withstand high stress. This structural resilience makes wood a versatile material for applications ranging from construction to advanced composites. However, a detailed understanding of how delignification affects softwood tracheid and hardwood fiber morphology is crucial for predicting material behavior and developing modified wood products. This study investigated the overall structural changes due to delignification, in five wood species, namely, spruce, beech, balsa, Douglas fir, and poplar. It additionally provides detailed morphology of delignified single tracheids and fibers. Scanning electron microscopy was used to compare the morphology between untreated and delignified fibers and tracheids. X-ray tomography enabled us to reconstruct high-resolution 3D models of delignified single tracheids or fibers, providing information on the pit arrangements. Moreover, delignification resulted in facilitated separation of fibers and tracheids and frayed wall appearance. We observed similar tracheid/fiber diameters and wall thicknesses for all five wood species. These findings enhance our understanding of the wood fiber and tracheid structures across species and the effects of delignification. The 3D models provide a valuable resource for (1) understanding interspecies differences of fibers and tracheids, (2) optimizing the use of delignified wood in industrial applications (including bio-based and bio-inspired materials), and (3) physical modeling of wood regarding questions of wood biomechanics and water management.

## Introduction

Wood fibers in hardwood and tracheids in softwood play a crucial role in the structure and function of vascular plants, particularly in water conduction and mechanical support [1–2]. They are especially important in gymnosperms, where they serve as primary conduits for water transport, whereas in angiosperms, they function alongside vessel elements, contributing to both axial and lateral water movement by providing strength of the tissue [3–4]. Tracheids are specialized cells that are elongated, hollow, and tapered at both ends, forming an interconnected system that facilitates the transport of water and nutrients from the roots to the aerial parts of the plant [3]. Depending on the species and function, conifer tracheids are generally narrow, with varying diameters (8–80 µm) [5]. Overall, vessels and fibers of angiosperms are less uniform with higher ranges of length than those of gymnosperm tracheids, whereas gymnosperm tracheids have similar diameters [3,6].

An important feature of vessel elements and tracheids is the presence of pits in the cell wall, where water moves between adjacent fibers and tracheids. Pits are distinguished between simple pits and bordered pits, which are surrounded by a thickened rim of wall material [6–7]. Several plants, such as conifers, have a torus–margo structure with a thin, porous mesh-like region (margo) and a thickened central part of the membrane (torus) that is slightly larger than the pit aperture [4,7].

Similar to other plant cells, fibers and tracheids have a primary wall (P) composed of cellulose, hemicelluloses, and pectin that are formed during cell growth [8–9]. Once fully grown, they develop two secondary walls (S1 and S2) and a tertiary wall (T) strongly supplemented by lignin. Apart from the middle lamella (ML) and the primary wall (P), the S2 layer being the thickest of the three main layers (S1, S2, and T), is the most important for mechanical support [1,10–11]. Lignin is present in all layers of the secondary and tertiary wall structure, with varying distribution and concentration across these layers but the highest proportions generally found in the S2 and T layers (spruce earlywood: ML 26.8%, S1 10.4%, S2+T 62.8%; spruce latewood: ML 18.4%, S1 7.9%, S2+T 73.7%) [12–13]. Moreover, lignification plays a key role in preventing cell collapse under high tensile stress or/and inner pressure exerted by the water column during transpiration [8].

Wood delignification is a crucial process in various industries, particularly pulp and paper production, biofuel generation, and the development of advanced wood-based materials [11,14]. This process involves the removal of lignin, which can significantly alter the properties of wood, making it more porous, flexible, and amenable to further processing [11]. Several methods have been employed for wood delignification, including chemical, enzymatic, and biological approaches [11,15–16]. The applications of delignified wood are diverse and expanding; they include, for example, the production of high-quality paper or of nanocellulose with applications in electronics, biomedical devices, and advanced composites [11,14]. Recent research has focused on developing more environmentally friendly and efficient delignification methods [17], as well as exploring novel applications for delignified wood in fields such as energy storage, water purification, and sustainable packaging materials [11,18–19].

Given the high versatility of wood across a wide range of applications [18,20–21], investigating the hierarchical structures in fibers and tracheids across different wood species and treatments is essential. By studying delignified fibers and tracheids, one can gain insights into the specific roles of lignin in maintaining tracheid shape, arrangement, and connections between neighboring cells. This knowledge is valuable for optimizing wood processing techniques, developing new wood-based materials and improving the understanding of wood anatomy and its biological function. By removing lignin, it is now possible to obtain high-resolution 3D models of separated single fibers and tracheids, revealing previously hidden details about pit arrangements and morphological variations across different wood species.

Despite the importance of wood fibers and tracheids in structure and function of wood, detailed 3D models of individual fibers and tracheids have been largely absent from scientific literature. Although previous studies have examined wood anatomy using various imaging techniques, such as X-ray micro-computed tomography in addition to scanning and transmission electron microscopy [22–27], high-resolution 3D reconstructions of single fibers and tracheids across multiple species are lacking. This knowledge gap has limited our understanding of the fine structural details and variations in tracheid/fiber morphology.

Therefore, we compared the morphological differences between the fibers of hardwood and tracheids of softwood of five wood species in this study by observation of the structural differences after delignification. Using X-ray tomography, we were able to build 3D models of single fibers and tracheids. Additionally, we imaged untreated and delignified samples using scanning electron microscopy for measuring fiber and tracheid diameter and wall thickness. The 3D models presented in this study provide novel insights into the tracheid/fiber shape, pit arrangements, and structural changes after delignification at an unprecedented level of detail. These results fill the needs for new wood anatomy research and provide further insights for applications in computer modelling of mechanical properties of fibers and tracheids and for development of novel bio-inspired materials for the industry.

## Material and Methods

### Wood samples

Wood samples of five different species were used in this study, namely, spruce (*Picea abies* (L.) H.Karst*),* beech *(Fagus sylvatica* L.*),* balsa *(Ochroma pyramidale* (Cav. ex Lam.) Urb.)*,* Douglas fir *(Pseudotsuga menziesii* (Mirbel) Franco), and poplar *(Populus* spp. L.). The wood samples were obtained from a local DIY supplier (Lower Bavaria, Germany). Wood identity was ensured by stereomicroscopical investigation of basic anatomical wood features. The samples were cut according to the main anatomical directions for further use. Care was taken that representative areas for each wood species could be investigated (softwoods: earlywood and latewood regions; hardwood: libriform basic tissue).

### Sample preparation

Untreated samples were cut into smaller pieces (1 cm × 1 cm × 1 cm) without further treatment. For the treated samples, prior to delignification, wood was prepared by Soxhlet extraction, as previously reported [28], with minor modifications. The wood samples were treated with two consecutive extractions, first using distilled water and acetone (1:6, v/v, ≥99.5%, Carl Roth, Karlsruhe, Germany), followed by toluene (≥99.5%, Carl Roth) for 24 h each. Afterwards, toluene was washed out by extraction with ethanol for 2 h, and the samples were dried at reduced pressure overnight (15 mbar, 40 °C).

For delignification, the wood specimens were added to a solution of NaClO_2_ (10% (w/v), AppliChem, Darmstadt, Germany) at 75 °C. The reaction was initiated by the addition of glacial acetic acid (4.0% (w/v), 100% purity, VWR) and maintained at this temperature for 6 h, during which the color of the solution changed from dark brown to yellow. The procedure was repeated once for 8 h to ensure full delignification of the wood, as indicated by the complete loss of color of the specimen. The wood pieces were washed with Soxhlet extraction using distilled water as solvent for 5 h, followed by freezing in liquid nitrogen and subsequent lyophilization overnight (−45 °C, 0.080 mbar, Christ Alpha 2–4 LDplus, Christ, Osterode, Germany).

### Scanning electron microscopy

Air-dried wood samples of each of the five species (untreated and delignified) were glued onto metal (SEM) stubs and sputter-coated with a 10 nm layer of gold–palladium (Leica Bal-TEC SCD500, Leica, Wetzlar, Germany). The samples were then visualized using a scanning electron microscope (SEM) Hitachi S-4800 (Hitachi, Tokyo, Japan) at an accelerating voltage of 1.5 kV.

### Nanotomography and 3D reconstruction

Single fibers and tracheids were easily mechanically separated from the delignified bulk samples without damaging them using fine tweezers and glued on top of conical sample holders using polyvinyl siloxane (President Light Body, Coltene, Altstätten, Switzerland). The dry fibers and tracheids were imaged (at 22.5 °C and 25% RH) using the X-ray tomography setup at the nanotomography endstation of beamline P05 of PETRA III at Deutsches Elektronen-Synchrotron (DESY). The X-ray beam was monochromatized using a Si(111) double crystal monochromator at an energy of 11 keV with a Zernike phase contrast [29]. An X-ray sCMOS camera (Hamamatsu C12849-101U, Pdet = 6.5 µm pixel size, 2048 × 2048 pixel, 16 bit image depth) with a 10 µm Gadox scintillator was used as the detector. For high-contrast and low-dose imaging, holotomography was applied as the phase contrast technique. Here, a gold Fresnel zone plate with a diameter of 300 µm was used [30]. By varying the sample-to-detector distance, different magnifications with fields of view ranging from 48 × 48 µm^2^ to 320 × 320 µm^2^ and effective pixel sizes of the raw data from 21.6 nm to 157.3 nm were used. At least two samples of each delignified wood species were scanned, resulting in a total of 35 tomograms. The most representative examples without obvious deformations (16 tomograms: spruce *n* = 4, beech *n* = 2, balsa *n* = 4, Douglas fir *n* = 2, and poplar *n* = 4) were selected for presentation.

The phase retrieval was performed using HoloTomoToolbox [31], while the tomographic reconstruction was performed using the P05 reconstruction pipeline, which is based on tomopy [32], as described earlier [30]. The most representative examples (4× spruce, 2× beech, 4× balsa, 2× Douglas fir, and 4× poplar) were then segmented manually with the brush tool and semi-automated with the magic wand tool using Amira 6.0.1 software (FEI SAS, Lyon, France). The segmented data were further processed in Blender software (Blender Foundation, Amsterdam, Netherlands, https://www.blender.org).

Additional interactive 3D models of two samples for each species are available online with links for each model in Supporting Information File 1.

### Estimation of tracheid/fiber diameter and wall thickness

Tracheid/fiber diameter and wall thickness were measured from the nanotomography reconstructions using ImageJ software (version 1.53, National Institute of Health, Bethesda, USA [33]). The raw scans were transferred to ImageJ, and the pixel sizes were set based on the respective effective pixel size of the scan. Because the diameters differed strongly within and between several wood species, we measured two diameters of each tracheid and fiber, that is, the long and short axes of the lumen from cross section per sample and taking both diameters two times per sample (see Supporting Information File 1, Figure S1). This resulted in spruce: *n* (number of individual samples) = 8 per diameter; beech: *n* = 4 per diameter; balsa: *n* = 8 per diameter; Douglas fir: *n* = 4 per diameter; and poplar: *n* = 8 per diameter. Differing sample size resulted from the quality of the scans, as some scans were not of sufficient quality for the purpose of this study. We therefore only worked with the scans with sufficient quality (16 in total).

Moreover, the diameter and wall thickness were additionally measured from SEM images of the untreated and delignified samples (always *n* = 8 per diameter). For the whole experimental setup and a scheme for the measurements, see Supporting Information File 1, Figure S1.

Statistical analyses were performed using RStudio (version 2024.09.0+375). Since the data was not normally distributed, the median values among the groups were compared using Kruskal–Wallis one-way analysis of variance (ANOVA) on ranks and further pairwise comparisons with Dunn’s post-hoc test and Holm’s correction, with α = 0.05 for each statistical test. Due to the relatively small and varying sample size, we decided to only use the unadjusted p-values for our statistical comparisons [34], but both unadjusted and adjusted p-values, can be found in Supporting Information File 1, Table S1 and Table S2.

## Results

### Morphologies of untreated and delignified fibers and tracheids of five wood species

**Figure 1:**
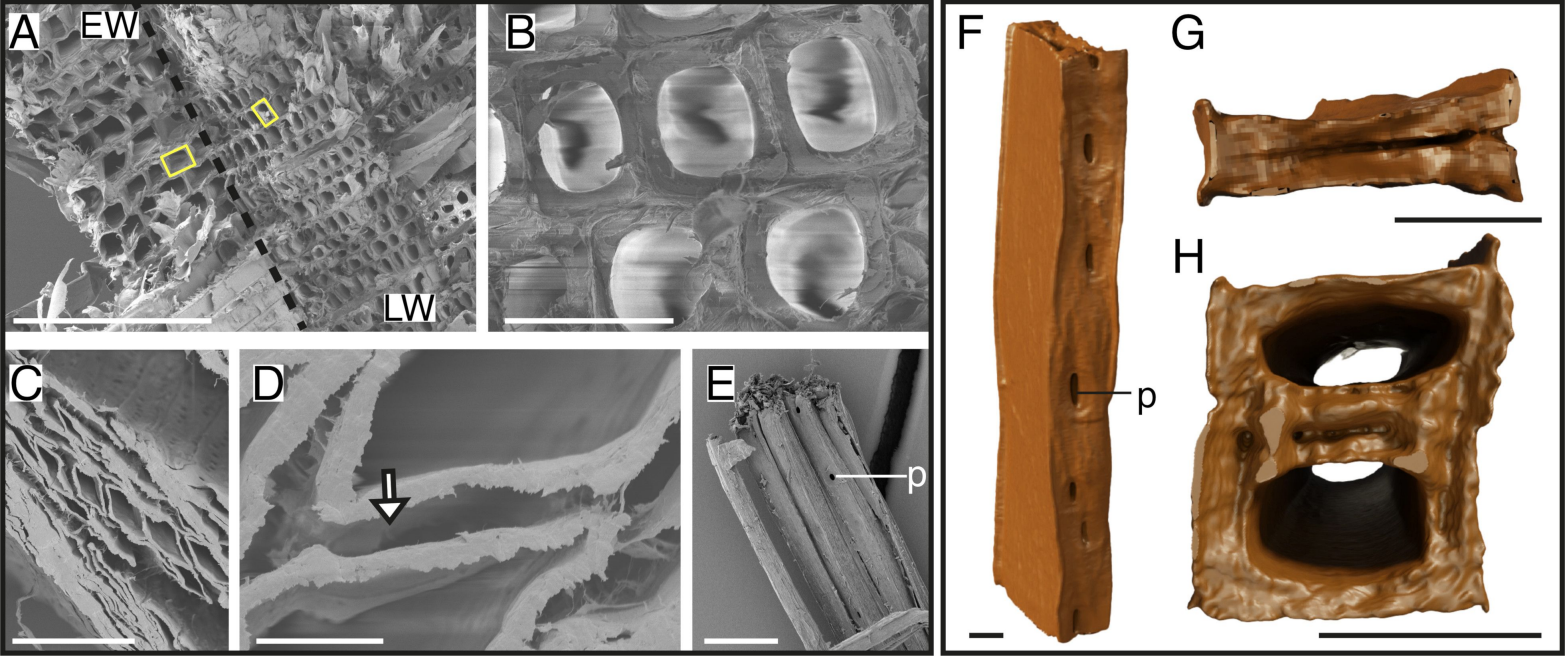
Structure of spruce tracheids. SEM images of untreated and delignified spruce (A–E) and 3D models of delignified tracheids (F–H). Untreated spruce samples (A,B) with larger tracheids (yellow rectangle; EW, left side), smaller tracheids (yellow rectangle; LW, right side), separated with a dotted line (black) (A), and connections between tracheids (B). Delignified spruce samples show a loosened structure (C), spaces between the walls of the tracheids (D, white arrow), and several tracheids with bordered pits (surrounded by a thickened rim of wall material) (E). Single delignified tracheid of LW in longitudinal view with pits on one side (F) and cross sections (G,H), which show the rectangular shape of a single tracheid of LW (G) and two neighboring tracheids of EW (H). Abbreviations: EW – earlywood, LW – latewood, p – pits. Scale bars: A – 300 µm; B – 30 µm; C – 100 µm; D – 10 µm; E – 50 µm; F, G, H – 10 µm.

**Figure 2:**
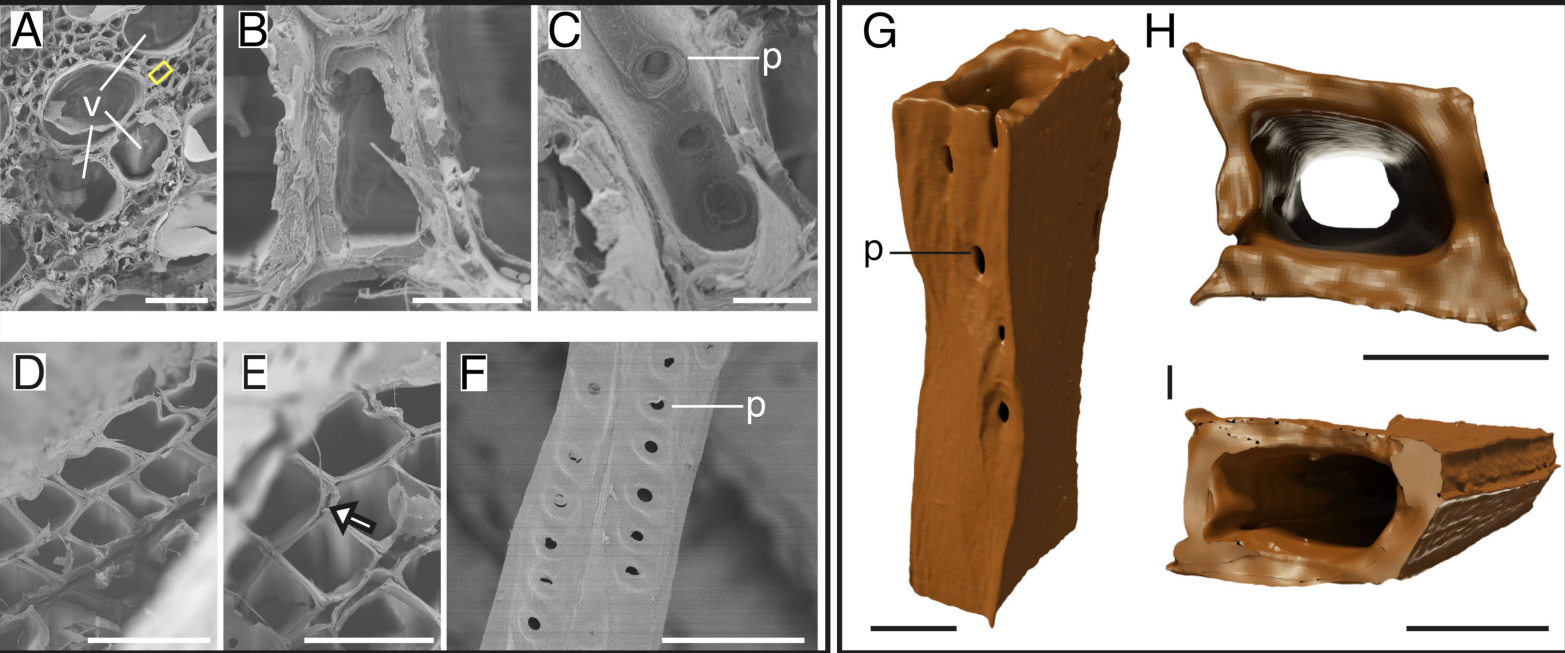
Structure of beech fibers. SEM images of untreated and delignified beech (A–F) and 3D models of delignified fibers (G–I). Untreated beech samples (A–C) with rectangular fibers (yellow rectangles) surrounding the vessels (A), connections between fibers (B), and bordered pits from the inside of the fiber (C). Delignified beech samples showed a loosened structure (D), spaces between the walls of the fibers (E, white arrow), and two fibers with bordered pits (F). Single delignified fiber in longitudinal view with pits on one side (G) and cross-sections (H,I), which show the rectangular/square shape of two beech fibers. Abbreviations: p – pits, v – vessels. Scale bars: A, D, F – 50 µm; B, C – 5 µm; E – 40 µm; G, H, I – 10 µm.

**Figure 3:**
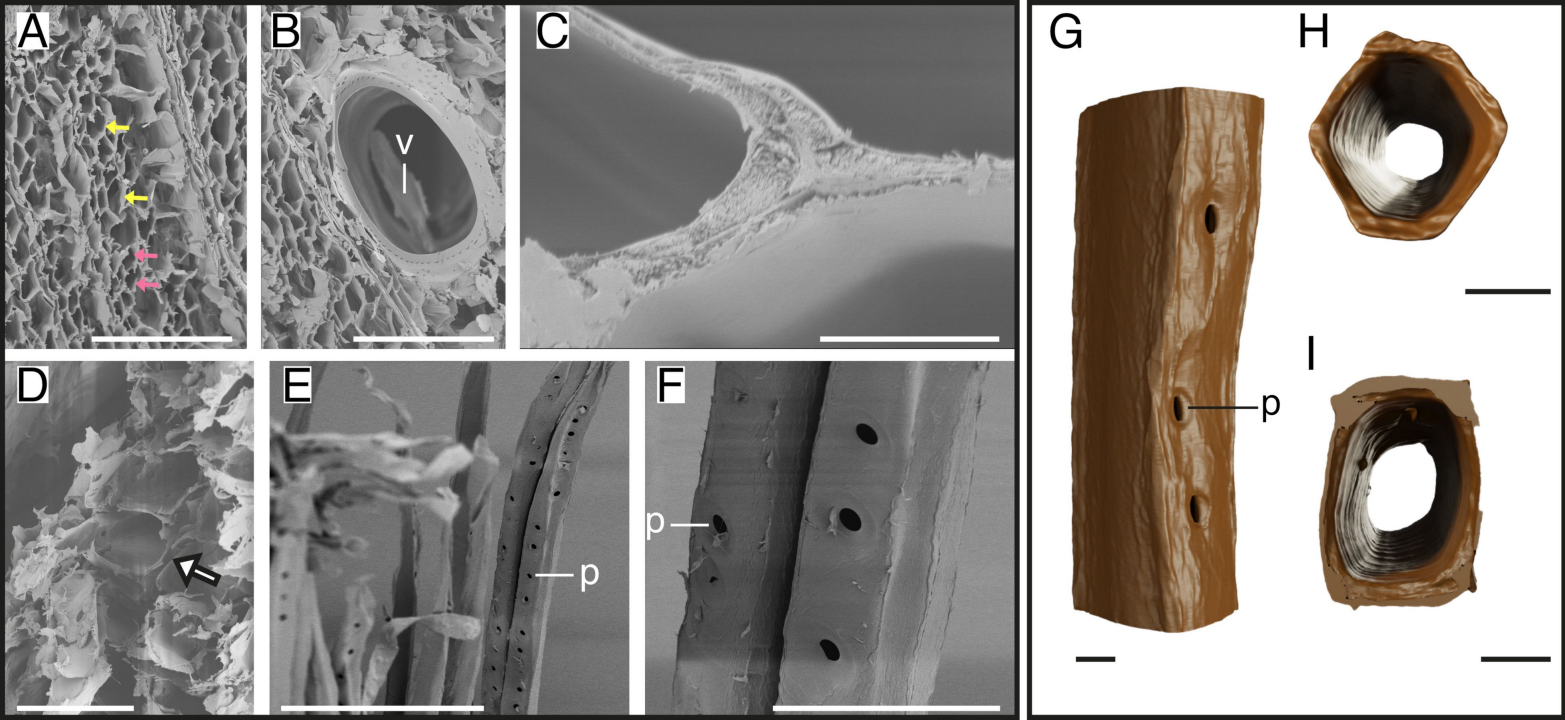
Structure of balsa fibers. SEM images of untreated and delignified balsa (A–F) and 3D models of delignified fibers (G–I). Untreated balsa samples (A–C) with fibers having hexagonal (yellow arrows) and rectangular (pink arrows) shapes (A), fibers surrounding the vessel (B), and close fiber connections (C). Delignified balsa samples show a loosened structure (D) with spaces between the walls of the fibers (white arrow) and fibers with bordered pits (E,F). Single delignified fiber in side view (G) and cross-sections (H,I) showing the hexagonal/rectangular shape of two balsa fibers and pits on one side of rectangular shape (G). Abbreviations: p – pits, v – vessels. Scale bars: A, B, E – 200 µm; C – 5 µm; D, F – 50 µm; G, H, I – 10 µm.

**Figure 4:**
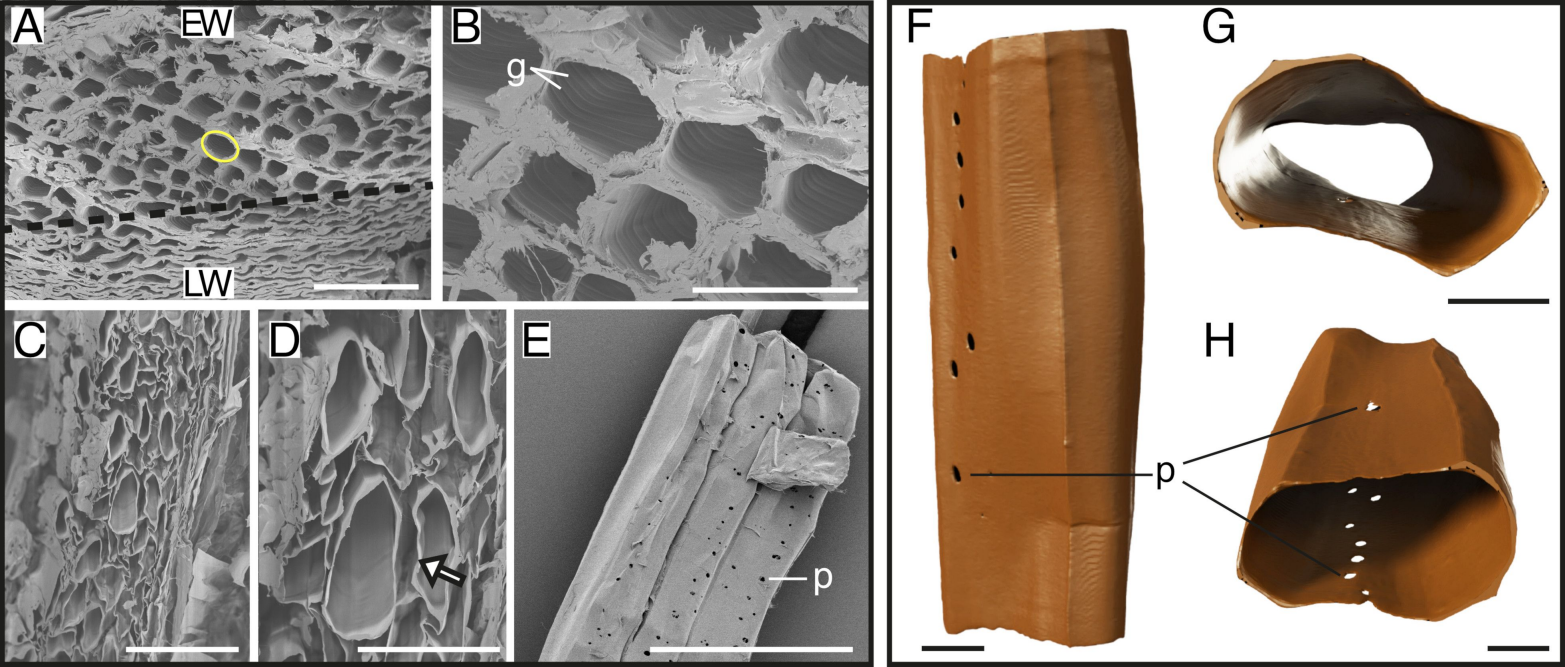
Structure of the Douglas fir tracheids. SEM images of untreated and delignified Douglas fir (A–E) and 3D models of delignified tracheids (F–H). Untreated Douglas fir samples (A,B) with tracheids in compressed (LW, lower part of the picture) and widened (EW, upper + center part of the picture) oval shape (yellow shape) (A), and connections between tracheids with special inner groove structures (B). Delignified Douglas fir samples showed a loosened structure (C) with spaces between the walls of the tracheids (D, white arrow) and tracheids with pits (E). Single delignified tracheid in longitudinal view with pits of EW (F) and cross sections of EW (G,H), which show the oval and bean shapes of two Douglas fir tracheids and pits on two sides (H). Abbreviations: g – grooves, p – pits. Scale bars: A, C, E – 100 µm; B – 40 µm, D – 50 µm; F, G, H – 10 µm.

**Figure 5:**
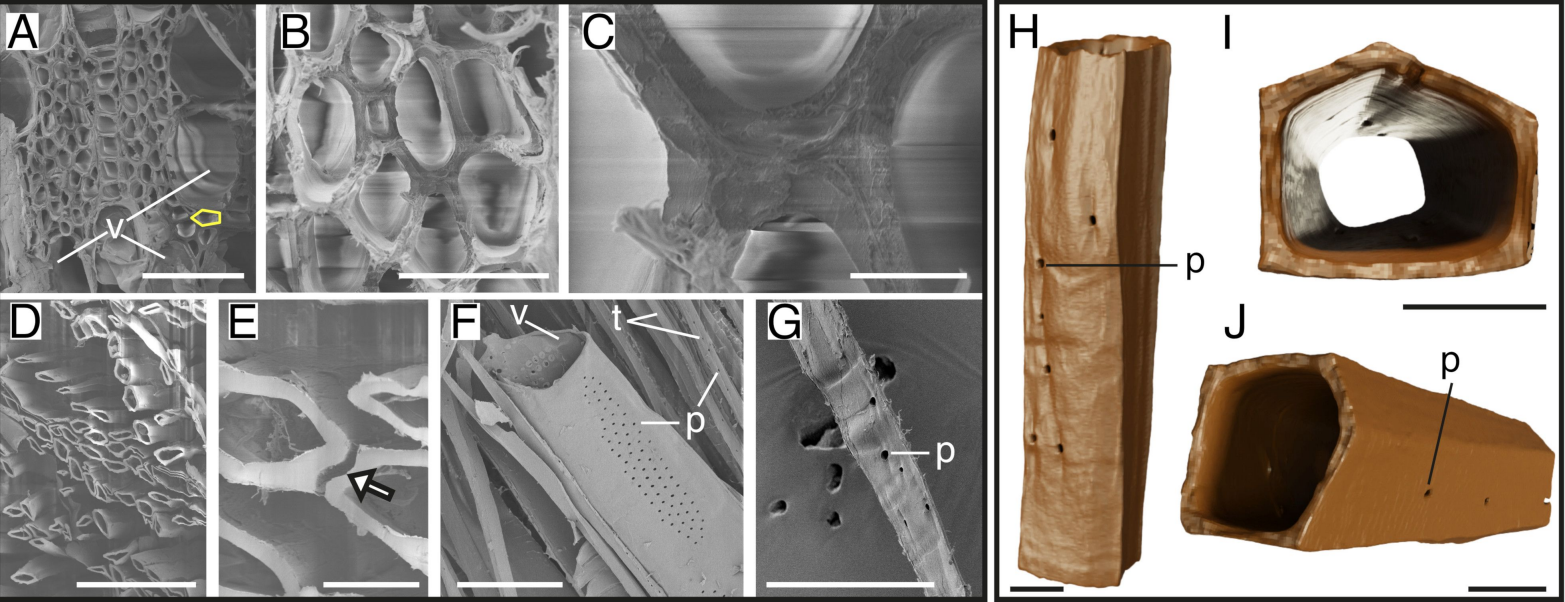
Structure of poplar fibers. SEM images of the untreated and delignified poplar (A–G) and 3D models of delignified fibers (H–J). Untreated poplar samples (A–C) with fibers (one example marked in yellow) surrounded by vessels (A) and connections between fibers of different sizes (B,C). The delignified poplar samples show a strongly loosened structure (D) with various spaces between the walls of the fibers (E, white arrow) and a large vessel with pits (F) as well as a fiber with pits (G). Single delignified fiber in longitudinal view with pits (H) and cross sections (I,J), which show the pentagonal shape of two poplar fibers and pits on one side. Abbreviations: p – pits, t – fibers/tracheids, v – vessels. Scale bars: A, D, F – 100 µm; B – 30 µm; C – 5 µm; E – 10 µm; G – 50 µm; H, I, J – 10 µm.

SEM images of the samples of each wood species are shown below in Figures 1–5, enabling comparisons between the morphology of untreated and delignified fibers and tracheids. After delignification, the single tracheids/fibers could easily be extracted and prepared for tomography. Using the reconstructed data, we built 3D models of at least two samples for each wood species. The shape of the fibers and tracheids and pit arrangements of each wood species are presented alongside the SEM images of untreated and delignified fibers and tracheids.

In untreated spruce, both earlywood (EW) and latewood (LW) were visible, with tracheids in LW having a smaller lumen than those in EW (Figure 1A, yellow rectangles). Both showed close connections to the neighboring tracheids (Figure 1B). After delignification, the tracheids were separated and spaces between the neighboring tracheids were visible (Figure 1C,D). Moreover, the tracheid walls appeared frayed (Figure 1D,E). The tracheid showed a rectangular shape with round lumen in the untreated spruce (Figure 1B), while deformed to rhombus in the delignified spruce. Such deformation was very likely due to the mechanical stress applied on the sample during experimental preparation (Figure 1H). Also, we found rectangular single tracheids with almost no lumen, which were potentially taken from LW areas (Figure 1G). On one side of the tracheid, with a smaller diameter, the pits were arranged longitudinally and in one column (Figure 1F).

Beech fibers showed close connections with the vessels and neighboring fibers in the untreated samples (Figure 2A,B) with bordered pits longitudinally located on one side of the wall (Figure 2C). After delignification, the fibers were more separated, with small spaces between neighboring fiber walls (Figure 2D,E). Similar to the untreated fibers, the delignified fibers exhibited bordered pits on one side (Figure 2F). The fiber had a rectangular/square shape, but less uniform than that of spruce tracheids (Figure 2B,H,I). The pits were longitudinally located on one side of the fiber, with a slightly spiral arrangement (Figure 2G).

In the balsa fiber wood SEM images, we observed two shapes of fibers within a small area of the untreated sample, that is, rectangular and hexagonal ones (Figure 3A). In comparison to fibers, the vessels were larger and had thicker walls (Figure 3B). Similar to beech, the connections between fibers were close in balsa, but the overall wall thickness was smaller (Figure 3C). After delignification, the fibers began to separate from each other and showed spaces between fiber walls (Figure 3D). These spaces were also visible in the longitudinal images of several fibers, which showed bordered pits on the fiber sides (Figure 3E,F). In the tomography data, we found pits on one side of the rectangular balsa fibers (Figure 3G), whereas the hexagonal fibers did not exhibit pits. The 3D models show the same as the SEM images, that is, some fibers had a hexagonal shape (Figure 3H), while others showed a rectangular shape (Figure 3G,I).

Interestingly, untreated Douglas fir samples showed tracheids of different shapes. In one sample, we found rectangular tracheids with a wide lumen (EW), as well as oval and squeezed tracheids (LW) (Figure 4A). Moreover, the inner part of the untreated tracheid wall exhibited a structure with grooves (Figure 4B), which was not observed in delignified tracheids of Douglas fir from both tomography data and SEM images. After delignification, it seemed like the tracheids appeared to change in shape; here, we imaged more oval and bean-shaped tracheids with wide lumina (Figure 4C). Similar to the observations in all other wood species, the connections between the tracheids were lost, resulting in spaces between the tracheids (Figure 4D). In addition to the 3D models, SEM images showed a large number of small pits arranged longitudinally on the tracheids (Figure 4E). Differing from previous species, Douglas fir tracheids had oval or bean shapes, as well as a smaller ratio of tracheid diameter and wall thickness (Figure 4F–H). Besides, the pits of Douglas fir tracheids were smaller and less ordered, and one tracheid had more pits than the fibers and tracheids of other tree species (Figure 4F). In addition, one or maybe two pits were visible on the opposite side (Figure 4H).

Finally, the SEM images of untreated poplar samples showed differently arranged fibers surrounded by vessels (Figure 5A). The small fibers were closely connected to the larger fibers (Figure 5B,C). After delignification, the fibers were strongly separated and showed small to very large spaces and connection losses to neighboring fibers (Figure 5D,E). Vessels, which were also separated from fibers, showed bulks of pits on the walls, and neighboring fibers showed pits on the same sides (Figure 5F). In poplar, single fibers showed frayed outer walls, which was presumably due to delignification (Figure 5G). Additionally, an unusual pentagonal shape was observed here (Figure 5H–J). The pits were arranged longitudinally on one side of the fiber (Figure 5H,J).

### Comparisons between morphologies of the fibers/tracheids

**Figure 6:**
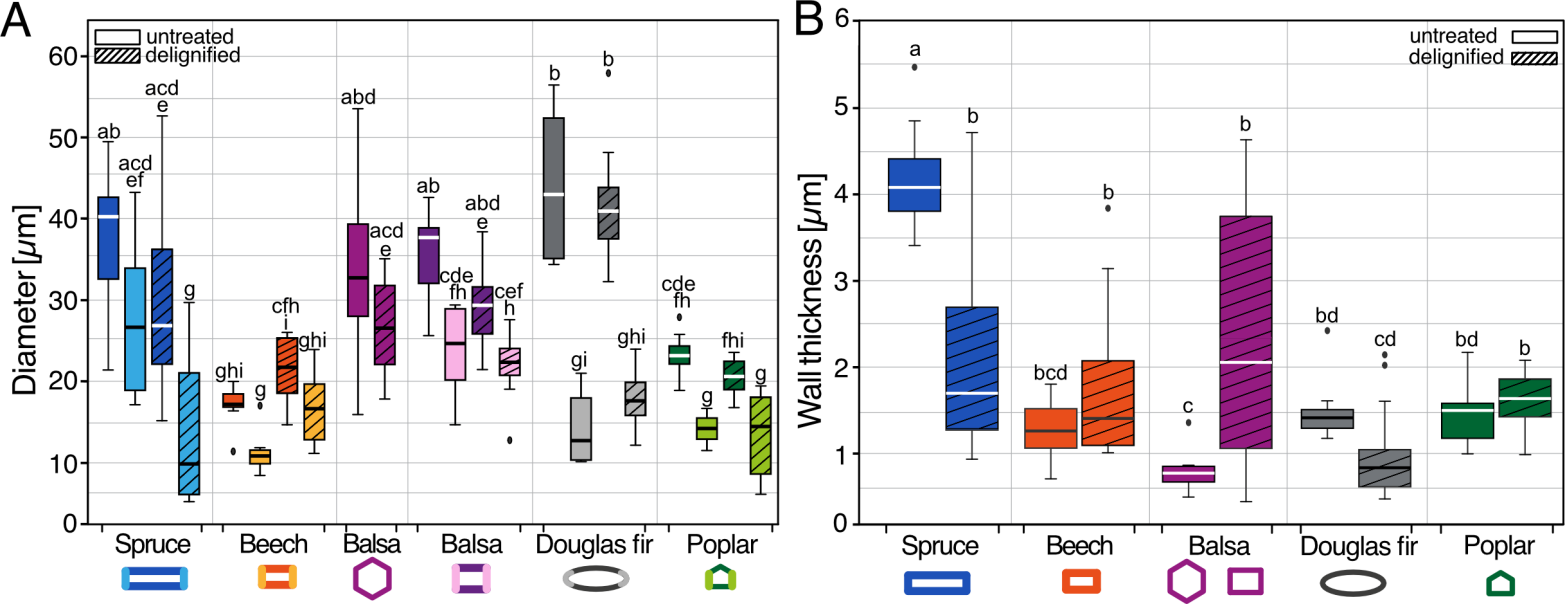
Fiber and tracheid diameter and wall thickness. Two fiber and tracheid diameters (A) of both untreated (plain boxes) and delignified (dashed boxes) wood samples. Diameter 1 in darker colors, and diameter 2 in lighter colors. For hexagonal fibers, only one diameter per treatment is provided, whereas for rectangular balsa, two diameters per treatment are measured. Tracheid and fiber wall thickness (B) of the untreated (plain boxes) and delignified (dashed boxes) wood samples are provided. Kruskal–Wallis ANOVA and Dunn’s post-hoc test were used for statistical processing of the data. Different letters indicate statistically significant differences. All statistically significant differences are presented in Supporting Information File 1, Tables S1 and S2. All untreated samples were *n* = 8, exception: balsa hexagonal *n* = 16. Delignified samples: diameter measurements: spruce and poplar *n* = 16, beech, balsa rectangular and Douglas fir *n* = 12, delignified balsa hexagonal *n* = 24; wall thickness measurements: spruce and poplar *n* = 16, beech and Douglas fir *n* = 12, balsa *n* = 22.

Because of the different tracheid and fiber shapes, we measured two diameters for each tracheid and fiber, namely, major and minor diameters. Here and below, the length of the long side is the major diameter (diameter 1), while that of the short side is the minor diameter (diameter 2). Since the balsa fiber had two shapes and the hexagonal shape exhibited uniform side length instead of two different lengths, we measured only one diameter for the hexagonal balsa fibers and reported it separately (Table 1). We found that within species, both diameters differed; also, the respective diameters differed after the treatment. For example, both diameters of the oval Douglas fir tracheids differed significantly in untreated and delignified samples (Kruskal–Wallis one-way ANOVA on ranks, *p* < 0.0001; Dunn’s post-hoc test, Douglas fir delignified diameter 1 vs diameter 2: *p* = 0.0001, Douglas fir untreated diameter 1 vs diameter 2: *p* = 0.0002; Figure 6A). In delignified spruce and poplar, the diameters differed significantly, as well as in untreated rectangular balsa fibers and untreated poplar (Kruskal–Wallis one-way ANOVA on ranks, *p* < 0.0001; for Dunn’s post-hoc test see Supporting Information File 1, Table S1). In addition, we compared the two diameters of untreated and delignified samples between species and found significant differences as well (Table 1, Figure 6A). Douglas fir had the overall largest tracheids among the five species (Table 1, Figure 6A) but showed similar diameters after delignification with a shrinkage of diameter 1 of 4.8% and an expansion of diameter 2 of 38.4%. In poplar, diameter 1 slightly shrunk by 10.7% and diameter 2 slightly expanded by 1.7%. Both diameters of spruce and balsa slightly decreased after delignification with significant differences of diameter 2 in spruce (Kruskal–Wallis one-way ANOVA on ranks, *p* < 0.0001; Dunn’s post-hoc test, *p* < 0.01) and with a shrinkage of diameter 1 of 33.3% and of diameter 2 of 63% in spruce. In balsa, diameter 1 shrunk by 22.1% and diameter 2 by 9.4%. In contrast, the diameters of beech slightly increased after delignification, but did not show any statistically significant difference with an expansion of diameter 1 of 26.5% and of diameter 2 of 52.5%. Moreover, diameters 1 of untreated Douglas fir and of untreated spruce were significantly larger than those of untreated beech and poplar (Kruskal–Wallis one-way ANOVA on ranks, *p* < 0.0001; Dunn’s post-hoc test, for p-values see Supporting Information File 1, Table S1; Figure 6A). Balsa showed the overall second largest diameters among the species, with a significantly larger diameter 1 of the untreated samples compared to the smallest in beech (Kruskal–Wallis one-way ANOVA on ranks, *p* < 0.0001; Dunn’s post-hoc test, *p* < 0.001), directly followed by spruce (Table 1, Figure 6A). The diameters of delignified spruce and of all beech and all poplar samples were in similar ranges (approximately 13–29 µm; Table 1, Figure 6A). All p-values for statistically significant differences are provided in Supporting Information File 1, Table S1. For the shrinking/expansion ratios of the diameters see Supporting Information File 1, Table S3.

**Table 1:** Comparison of the tracheid and fiber morphologies of the studied wood species. Shape, diameter, and wall thickness of the five wood species and the two treatments. Data shows mean ± standard deviation. All untreated samples were *n* = 8, exception: balsa hexagonal *n* = 16. Delignified samples: diameter measurements: spruce and poplar *n* = 16, beech, balsa rectangular and Douglas fir *n* = 12, delignified balsa hexagonal *n* = 24; wall thickness measurements: spruce and poplar *n* = 16, beech and Douglas fir *n* = 12, balsa *n* = 22.

Species	Treatment	Shape	Diameter [µm]		Wall thickness [µm]

			Diameter 1	Diameter 2	

spruce (*P. abies*)	untreated	rectangular	37.2 ± 9.9	27.4 ± 9.9	4.2 ± 0.6
	delignified		29.9 ± 11.4	13.1 ± 8.5	2.2 ± 1.2

beech (*F. sylvatica*)	untreated	rectangular	17.1 ± 2.6	11.3 ± 2.6	1.3 ± 0.4
	delignified		21.5 ± 4.0	16.6 ± 4.2	1.8 ± 0.9

balsa (*O. pyramidale*)	untreated	hexagonal	33.6 ± 8.9		0.8 ± 0.3
	delignified		27.2 ± 5.6		2.4 ± 1.5

	untreated	rectangular	35.8 ± 5.6	23.6 ± 5.9	0.8 ± 0.3
	delignified		29.2 ± 4.6	22.2 ± 3.9	2.4 ± 1.5

Douglas fir (*P. menziesii*)	untreated	oval	44.2 ± 9.2	14.3 ± 4.6	1.5 ± 0.4
	delignified	oval	41.7 ± 6.7	17.9 ± 3.6	1.0 ± 0.5

poplar (*P*. spp.)	untreated	pentagonal	23.2 ± 3.0	14.1 ± 1.8	1.5 ± 0.4
	delignified		20.7 ± 2.1	13.5 ± 5.1	1.6 ± 0.4

Since the wall thickness was homogenous within the species, we reported only one value for each species and treatment (Table 1, Figure 6B). No significant differences were found in the wall thickness between treatments of most examined tree species, except for balsa and spruce. In balsa, we observed that delignification significantly increased the wall thickness in comparison to the untreated balsa samples with an expansion of 165.8% (Kruskal–Wallis one-way ANOVA on ranks, *p* < 0.0001; Dunn’s post-hoc test, *p* < 0.009; Table 1, Figure 6B). In contrast, the wall thickness significantly decreased in spruce after delignification with a shrinkage of 58.4% (Kruskal–Wallis one-way ANOVA on ranks, *p* < 0.0001; Dunn’s post-hoc test, *p* < 0.05; Table 1, Figure 6B). When comparing untreated samples, spruce had overall the highest value for wall thickness and significantly higher than all other wood species (Kruskal–Wallis one-way ANOVA on ranks, *p* < 0.0001; Dunn’s post-hoc test, for p-values see Supporting Information File 1, Table S2; Table 1, Figure 6B). Comparing delignified samples, balsa and spruce had a significantly larger thickness than beech, Douglas fir, and poplar (Kruskal–Wallis one-way ANOVA on ranks, *p* < 0.0001; Dunn’s post-hoc test, for p-values see Supporting Information File 1, Table S2; Table 1, Figure 6B). All p-values for statistically significant differences are provided in Supporting Information File 1, Table S2. For the shrinking/expansion ratio of wall thickness see Supporting Information File 1, Table S4.

## Discussion

The results presented in this study provide distinct insights into the structural differences in wood fibers and tracheids across various species before and after delignification and novel data on high-resolution 3D morphology of single delignified fibers and tracheids. Tracheid and fiber shapes can vary strongly across the five tree species studied. We identified four distinct shapes, namely, rectangular tracheids and fibers in spruce (Figure 1), beech (Figure 2), and balsa (Figure 3), oval tracheids in Douglas fir (Figure 4), pentagonal fibers in poplar (Figure 5), and hexagonal fibers in balsa (Figure 3). The presence of two fiber shapes in balsa sets it apart from the other species, which exhibited only one shape. However, variations in lumen size are evident within softwood, as seen in the smaller lumina of latewood (LW) tracheids and the wider lumina of earlywood (EW) tracheids in both spruce (Figure 1A) and Douglas fir (Figure 4A).

Such variation in lumen size may reflect the evolutionary adaptations of different tree species to specific environmental conditions and their different functional requirements. For instance, larger lumina typically form in earlywood during optimal environmental conditions, while latewood tracheids develop narrower lumina as a response to limited growth resources [35–36]. It seems that larger lumina in earlywood enhance water transport efficiency during growth seasons, enabling the trees to support rapid growth. Conversely, narrower lumina in latewood provide superior mechanical support during periods of reduced growth, ensuring structural stability. However, across the five species studied here, the hardwood fibers, in general, show wider lumina than softwood tracheids. Environmental factors, such as higher environmental temperatures and less compact, well-aerated soils also promote the development of fibers and tracheids with larger lumina [37–38], which very likely result from high evaporation, as well as suitable environmental conditions for growth. In brief, tracheid dimensions are not static; they vary within individual trees or between species and are influenced by multiple factors, including environmental conditions and seasonal growth patterns [37,39].

Using 3D models, we additionally showed pit arrangements of single fibers and tracheids with unprecedented 3D details in spatial distribution compared to traditional methods such as SEM, which, however, show more details of the pit structures [40–42]. Unlike those conventional imaging methods, nano-CT 3D models provide a comprehensive view of pit distribution and spatial organization, enabling a deeper understanding of their role in water transportation and potential mechanical side effects [25–27]. However, due to the small sample size, we can only speculate on the real pit distribution and only show an insight into a small number of 3D-reconstructed fibers and tracheids. To make better conclusions about the pit distribution, a higher sample size and results from several methodologies are needed. In our data, we found that the pits could be located either on only one side of the tracheids and fibers, for example, in spruce (Figure 1), beech (Figure 2), or on multiple sides of the fibers, for example, in balsa (Figure 3) and poplar (Figure 5), which does not correspond with the descriptions of Richter and colleagues [43]. Usually, beech has pits on tangential and radial walls, while fiber pits of poplar appear only on radial walls [43]. In Douglas fir, the pits were generally smaller, more frequent and less ordered compared to the other four species (Figure 4). Moreover, the pit arrangements and distributions vary with tree age and tracheid/fiber characteristics. For instance, in *Pinus radiata*, the number of pits per tracheid is related more to tracheid length in the first ten years of growth, whereas it primarily correlates with wood age [44]. Additionally, tracheid diameter and pit diameter can have a positive linear relationship, with larger fibers or tracheids containing larger pits [44–45]. This complies with our observation when comparing spruce and balsa samples to the poplar samples. The functional implications of pit arrangement for water transportation across species remain unclear. Nevertheless, it is worth mentioning the general climatic backgrounds of the five species as this provides an ecological context for interpreting their wood anatomical features. In general, all five species in this study prefer humid and moist environments. Spruce and beech generally favor cool temperatures and adequate rainfall during growth season [46–47], Douglas fir and poplar perform better with moderate temperatures, but are able to adapt to various environments [48–49], while balsa trees favor tropical and subtropical climates [50].

Across all wood types studied, delignification results into (1) facile separation of fibers and tracheids with visible spaces between neighboring walls (removal of the lignin-rich middle lamella) and (2) a frayed appearance of tracheid/fiber walls. These observations underline the crucial role of lignin in maintaining the structural integrity and cohesion between fibers and tracheids in wood [51–52]. In contrast to the overall arrangement changes of the wood after delignification, we did not observe significant dimensional changes of the fibers or tracheids. While Bao et al. [53] measured a decrease in wall thickness after delignification, our results showed that the wall thickness remains mostly unchanged after delignification, except for balsa, where wall thickness increased after delignification (Figure 6B). This increase might be due to differences in wood samples and possible discrepancies between EW and LW. The stability of the tracheid/fiber dimensions after delignification suggests that hemicellulose and cellulose may play an important role in maintaining the basic single tracheid/fiber structure after removing lignin [54–55]. This is also reasonable from a materials perspective since the elastic modulus of cellulose is 15 times that of lignin [56–57]. Moreover, lignin might contribute more to the bonding of fibers or tracheids instead of increasing the stability of a single tracheid since the wall thickness remained the same after delignification, but spaces between fibers and tracheids appeared. In addition, gaps between adjacent fibers and tracheids after delignification have also been reported previously [41,43]. Overall, by delignification of the samples and the following nanotomography, we were able to show 3D models of single tracheids and fibers, by easily separating them without further destruction of the tracheids or fibers and whole samples. We were able to show models of neatly separated tracheids and fibers with clean walls. Using nanotomography in combination with delignified samples can improve our understanding of tracheid/fiber and pit distribution of single cells, which was not possible to differentiate and separate in previous tomographical imaging studies [41].

This study represents a high-resolution 3D imaging approach to delignified single fibers and tracheids, paving the way for more effective modeling applications, such as finite element modeling or computational simulations of fluid flow or heat transfer, aiding in development of various industrial applications of delignified wood. Accurate 3D models provide valuable insights into tracheid and fiber morphology, offering implications for wood processing techniques and the development of innovative wood-based materials, including bioinspired materials. This study also holds the potential for shedding light on evolutionary adaptations and taxonomic relationships among species. Still, as the sample size was relatively small, our results show a trend. As wood is a highly variable material depending on the growth conditions, further studies with higher sample sizes could help in verifying those trends or showing discrepancies to samples with differing growth conditions.

In conclusion, this study demonstrates the diverse structural adaptations of wood fibers and tracheids across different tree species and the critical role of lignin in maintaining the wood structure. These findings have implications for understanding wood properties, processing techniques, and potential applications in various branches of industry.

## Supporting Information

File 1 Additional experimental data.

## Data Availability

Data generated and analyzed during this study is available from the corresponding author upon reasonable request. The tomography data were uploaded to Sketchfab. The corresponding URLs are provided in Supporting Information File 1 of the article.
