# Supplementary material for: Comparative study on 3D morphologies of delignified, single tracheids and fibers of five wood species
Source: Beilstein J Nanotechnol. 2026 Feb 4;17:239–50. doi: 10.3762/bjnano.17.16 (PMC12884549; doi:10.3762/bjnano.17.16)
Supplement: File 1 — Additional experimental data. [file Beilstein_J_Nanotechnol-17-239-s001.pdf]

BEILSTEIN JOURNAL OF NANOTECHNOLOGY

Supporting Information

for

Comparative study on 3D morphologies of delignified, single tracheids and fibers of five wood species

Helen Gorges, Felicitas von Usslar, Cordt Zollfrank, Silja Flenner, Imke Greving, Martin Müller, Clemens F. Schaber, Chuchu Li and Stanislav N. Gorb

Beilstein J. Nanotechnol. **2026,** *17,* 239–250. doi:10.3762/bjnano.17.16

Additional experimental data

License and Terms: This is a supporting information file under the terms of the Creative Commons Attribution License (https://creativecommons.org/licenses/by/4.0). Please note that the reuse, redistribution and reproduction in particular requires that the author(s) and source are credited and that individual graphics may be subject to special legal provisions.
The license is subject to the *Beilstein Journal of Nanotechnology* terms and conditions: (https://www.beilstein-journals.org/bjnano/terms)

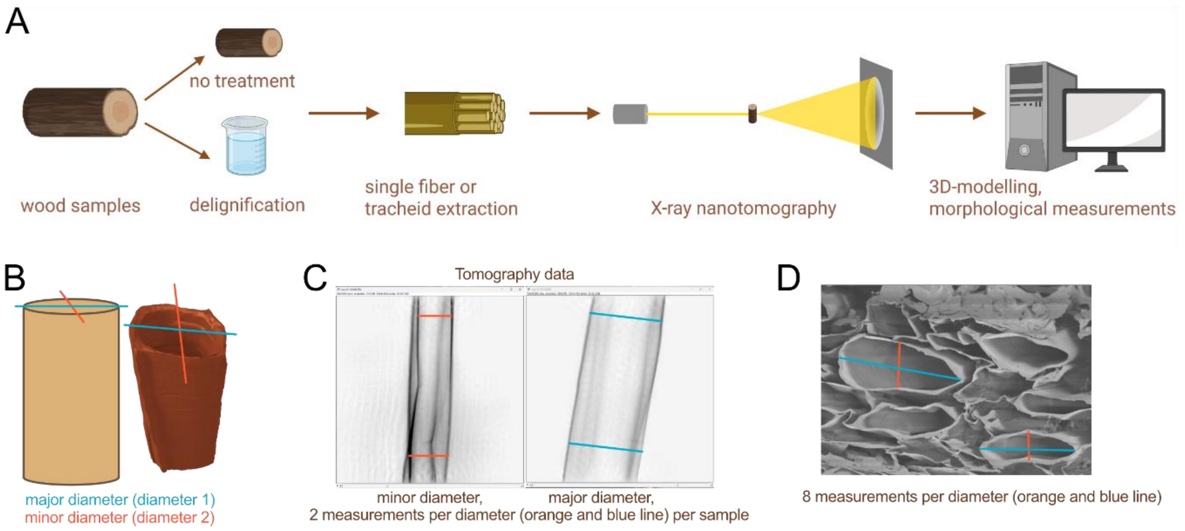

Figure S1: Experimental setup and diameter measuring method. (A) Experimental setup, from wood sample to either no treatment or delignification. Then single fiber/tracheid extraction and X-ray nanotomography. As the last step 3D-modelling and morphological measurements on the tomography data and SEM images. Figure 1(A) was created in BioRender. Gorges, H. (2026) https://BioRender.com/qgpxmnp. This content is not subject to CC BY 4.0. (B) Direction of the two measured diameters with major diameter (diameter 1) in blue and minor diameter (diameter 2) in orange. (C) Diameter measurement of the tomography data. We measured 2 minor (orange) and 2 major (blue) diameters of each sample, resulting in: spruce major n = 8, minor n = 8; beech major n = 4, minor n = 4; balsa hexagonal major n = 4, minor n = 4; balsa rectangular major n = 4, minor n = 4; Douglas fir major n = 4, minor n = 4; poplar major n = 8, minor n = 8. (D) Diameter measurement of SEM images, with major diameter in blue and minor diameter in orange. We measured 8 major diameters per species (n = 8) and 8 minor diameters per species (n = 8).

Links for each 3D model:

Spruce 1: single tracheid
https://sketchfab.com/3d-models/spruce-1-single-tracheid-31a6667bfed049e583f55e4407602d26

Spruce 2: double tracheid
Https://sketchfab.com/3d-models/spruce-2-double-tracheid-e008a473c15a4305952ec82da82a9975

Beech 1: single fiber
https://sketchfab.com/3d-models/beech-1-single-fiber-24ae48dea5fd4d67bdade548ab6a5934

Beech 2: single fiber
https://sketchfab.com/3d-models/beech-2-single-fiber-994b14802e2c4ef5b7124e4d322f44cd

Balsa hexagonal: single fiber
https://sketchfab.com/3d-models/balsa-hexagonal-single-fiber-1eb70a22aab742ef961c55924d628202

Balsa rectangular: single fiber
https://sketchfab.com/3d-models/balsa-rectangular-single-siber-3bcfddb059fe4c6e9fbe7a18a3d95d37

Douglas fir 1: single tracheid
https://sketchfab.com/3d-models/douglas-fir-1-single-tracheid-6b7c7ddef0bd45b7b88daee08700f9bf

Douglas fir 2: single tracheid
https://sketchfab.com/3d-models/douglas-fir-2-single-tracheid-58d57fb0310246008ee4e6a4e7d59930

Poplar 1: single fiber
https://sketchfab.com/3d-models/poplar-1-single-fiber-e09cede049b0464f8f00174c13bbcc48

Poplar 2: single fiber
https://sketchfab.com/3d-models/poplar-2-single-fiber-3ccccd042b045bb83e1c84ef0fde816

Table S1: Statistical significances of tracheid diameters. Kruskal-Wallis ANOVA (p = 2.20E-16) with Dunn's post-hoc test and a Holm's correction.

Group 1	Group 2	Z	P.unadj	P.adj
Balsa hexagonal delignified	Balsa hexagonal untreated	-1.37303	1.70E-01	1.00E+00
	Balsa rectangular diameter 1 delignified	-0.63187	5.27E-01	1.00E+00
	Balsa rectangular diameter 1 untreated	-1.63751	1.02E-01	1.00E+00
	Balsa rectangular diameter 2 delignified	1.411762	1.58E-01	1.00E+00
	Balsa rectangular diameter 2 untreated	0.946946	3.44E-01	1.00E+00
	Beech diameter 1 delignified	1.742818	8.14E-02	1.00E+00
	Beech diameter 1 untreated	3.093082	1.98E-03	3.15E-01
	Beech diameter 2 delignified	3.550892	3.84E-04	6.64E-02
	Beech diameter 2 untreated	4.503163	6.69E-06	1.34E-03
	Douglas fir diameter 1 delignified	-2.66277	7.75E-03	1.00E+00
	Douglas fir diameter 1 untreated	-2.38597	1.70E-02	1.00E+00
	Douglas fir diameter 2 delignified	3.137072	1.71E-03	2.73E-01
	Douglas fir diameter 2 untreated	3.659595	2.53E-04	4.42E-02
	Poplar diameter 1 delignified	2.238688	2.52E-02	1.00E+00
	Poplar diameter 1 untreated	0.90973	3.63E-01	1.00E+00
	Poplar diameter 2 delignified	4.859208	1.18E-06	2.46E-04
	Poplar diameter 2 untreated	3.920109	8.85E-05	1.63E-02
	Spruce diameter 1 delignified	-0.12292	9.02E-01	1.00E+00
	Spruce diameter 1 untreated	-1.58376	1.13E-01	1.00E+00
	Spruce diameter 2 delignified	4.534912	5.76E-06	1.16E-03
	Spruce diameter 2 untreated	0.430054	6.67E-01	1.00E+00

Balsa hexagonal untreated	Balsa rectangular diameter 1 delignified	0.575421	5.65E-01	1.00E+00
	Balsa rectangular diameter 1 untreated	-0.52047	6.03E-01	1.00E+00
	Balsa rectangular diameter 2 delignified	2.467457	1.36E-02	1.00E+00
	Balsa rectangular diameter 2 untreated	1.916183	5.53E-02	1.00E+00
	Beech diameter 1 delignified	2.773956	5.54E-03	8.14E-01
	Beech diameter 1 untreated	3.939579	8.16E-05	1.51E-02
	Beech diameter 2 delignified	4.447907	8.67E-06	1.70E-03
	Beech diameter 2 untreated	5.269017	1.37E-07	3.02E-05
	Douglas fir diameter 1 delignified	-1.30483	1.92E-01	1.00E+00
	Douglas fir diameter 1 untreated	-1.22612	2.20E-01	1.00E+00
	Douglas fir diameter 2 delignified	4.064784	4.81E-05	8.99E-03
	Douglas fir diameter 2 untreated	4.473694	7.69E-06	1.52E-03
	Poplar diameter 1 delignified	3.29703	9.77E-04	1.63E-01
	Poplar diameter 1 untreated	1.881096	6.00E-02	1.00E+00
	Poplar diameter 2 delignified	5.689226	1.28E-08	2.90E-06
	Poplar diameter 2 untreated	4.719308	2.37E-06	4.90E-04
	Spruce diameter 1 delignified	1.141188	2.54E-01	1.00E+00
	Spruce diameter 1 untreated	-0.46979	6.39E-01	1.00E+00
	Spruce diameter 2 delignified	5.393186	6.92E-08	1.54E-05
	Spruce diameter 2 untreated	1.428853	1.53E-01	1.00E+00
Balsa rectangular diameter 1 delignified	Balsa rectangular diameter 1 untreated	-0.97519	3.29E-01	1.00E+00
	Balsa rectangular diameter 2 delignified	1.769838	7.68E-02	1.00E+00

	Balsa rectangular diameter 2 untreated	1.33642	1.81E-01	1.00E+00
Balsa rectangular diameter 1 delignified	Beech diameter 1 delignified	2.056541	3.97E-02	1.00E+00
	Beech diameter 1 untreated	3.255982	1.13E-03	1.86E-01
	Beech diameter 2 delignified	3.622379	2.92E-04	5.08E-02
	Beech diameter 2 untreated	4.517197	6.27E-06	1.26E-03
	Douglas fir diameter 1 delignified	-1.75881	7.86E-02	1.00E+00
	Douglas fir diameter 1 untreated	-1.64463	1.00E-01	1.00E+00
	Douglas fir diameter 2 delignified	3.264001	1.10E-03	1.82E-01
	Douglas fir diameter 2 untreated	3.762687	1.68E-04	2.96E-02
	Poplar diameter 1 delignified	2.477035	1.32E-02	1.00E+00
	Poplar diameter 1 untreated	1.303132	1.93E-01	1.00E+00
	Poplar diameter 2 delignified	4.691779	2.71E-06	5.58E-04
	Poplar diameter 2 untreated	3.995697	6.45E-05	1.20E-02
	Spruce diameter 1 delignified	0.481114	6.30E-01	1.00E+00
	Spruce diameter 1 untreated	-0.92711	3.54E-01	1.00E+00
	Spruce diameter 2 delignified	4.417699	9.98E-06	1.95E-03
	Spruce diameter 2 untreated	0.874097	3.82E-01	1.00E+00
Balsa rectangular diameter 1 untreated	Balsa rectangular diameter 2 delignified	2.558183	1.05E-02	1.00E+00
	Balsa rectangular diameter 2 untreated	2.110203	3.48E-02	1.00E+00
	Beech diameter 1 delignified	2.814618	4.88E-03	7.28E-01
	Beech diameter 1 untreated	3.862515	1.12E-04	2.03E-02
	Beech diameter 2 delignified	4.215146	2.50E-05	4.72E-03
	Beech diameter 2 untreated	5.013842	5.34E-07	1.15E-04

	Douglas fir diameter 1 delignified	-0.59794	5.50E-01	1.00E+00
Balsa rectangular diameter 1 untreated	Douglas fir diameter 1 untreated	-0.61111	5.41E-01	1.00E+00
	Douglas fir diameter 2 delignified	3.894603	9.84E-05	1.79E-02
	Douglas fir diameter 2 untreated	4.325072	1.52E-05	2.93E-03
	Poplar diameter 1 delignified	3.212482	1.32E-03	2.13E-01
	Poplar diameter 1 untreated	2.079816	3.75E-02	1.00E+00
	Poplar diameter 2 delignified	5.165703	2.40E-07	5.25E-05
	Poplar diameter 2 untreated	4.53778	5.68E-06	1.15E-03
	Spruce diameter 1 delignified	1.452245	1.46E-01	1.00E+00
	Spruce diameter 1 untreated	0.043892	9.65E-01	1.00E+00
	Spruce diameter 2 delignified	4.923987	8.48E-07	1.80E-04
	Spruce diameter 2 untreated	1.688162	9.14E-02	1.00E+00
Balsa rectangular diameter 2 delignified	Balsa rectangular diameter 2 untreated	-0.24657	8.05E-01	1.00E+00
	Beech diameter 1 delignified	0.286703	7.74E-01	1.00E+00
	Beech diameter 1 untreated	1.67299	9.43E-02	1.00E+00
	Beech diameter 2 delignified	1.852541	6.39E-02	1.00E+00
	Beech diameter 2 untreated	2.934205	3.34E-03	5.18E-01
	Douglas fir diameter 1 delignified	-3.52865	4.18E-04	7.14E-02
	Douglas fir diameter 1 untreated	-3.22763	1.25E-03	2.05E-01
	Douglas fir diameter 2 delignified	1.494162	1.35E-01	1.00E+00
	Douglas fir diameter 2 untreated	2.179695	2.93E-02	1.00E+00
	Poplar diameter 1 delignified	0.584999	5.59E-01	1.00E+00
	Poplar diameter 1 untreated	-0.27986	7.80E-01	1.00E+00

	Poplar diameter 2 delignified	2.799743	5.11E-03	7.57E-01
Balsa rectangular diameter 2 delignified	Poplar diameter 2 untreated	2.412706	1.58E-02	1.00E+00
	Spruce diameter 1 delignified	-1.41092	1.58E-01	1.00E+00
	Spruce diameter 1 untreated	-2.5101	1.21E-02	1.00E+00
	Spruce diameter 2 delignified	2.525663	1.15E-02	1.00E+00
	Spruce diameter 2 untreated	-0.70889	4.78E-01	1.00E+00
Balsa rectangular diameter 2 untreated	Beech diameter 1 delignified	0.503007	6.15E-01	1.00E+00
	Beech diameter 1 untreated	1.752312	7.97E-02	1.00E+00
	Beech diameter 2 delignified	1.903535	5.70E-02	1.00E+00
	Beech diameter 2 untreated	2.903639	3.69E-03	5.61E-01
	Douglas fir diameter 1 delignified	-2.90955	3.62E-03	5.54E-01
	Douglas fir diameter 1 untreated	-2.72132	6.50E-03	9.30E-01
	Douglas fir diameter 2 delignified	1.582991	1.13E-01	1.00E+00
	Douglas fir diameter 2 untreated	2.214869	2.68E-02	1.00E+00
	Poplar diameter 1 delignified	0.77583	4.38E-01	1.00E+00
	Poplar diameter 1 untreated	-0.03039	9.76E-01	1.00E+00
	Poplar diameter 2 delignified	2.729051	6.35E-03	9.15E-01
	Poplar diameter 2 untreated	2.427577	1.52E-02	1.00E+00
	Spruce diameter 1 delignified	-0.98441	3.25E-01	1.00E+00
	Spruce diameter 1 untreated	-2.06631	3.88E-02	1.00E+00
	Spruce diameter 2 delignified	2.487335	1.29E-02	1.00E+00
	Spruce diameter 2 untreated	-0.42204	6.73E-01	1.00E+00
Beech diameter 1 delignified	Beech diameter 1 untreated	1.416555	1.57E-01	1.00E+00

	Beech diameter 2 delignified	1.565838	1.17E-01	1.00E+00
Beech diameter 1 delignified	Beech diameter 2 untreated	2.677771	7.41E-03	1.00E+00
	Douglas fir diameter 1 delignified	-3.81535	1.36E-04	2.42E-02
	Douglas fir diameter 1 untreated	-3.48406	4.94E-04	8.40E-02
	Douglas fir diameter 2 delignified	1.20746	2.27E-01	1.00E+00
	Douglas fir diameter 2 untreated	1.923261	5.44E-02	1.00E+00
	Poplar diameter 1 delignified	0.278501	7.81E-01	1.00E+00
	Poplar diameter 1 untreated	-0.53629	5.92E-01	1.00E+00
	Poplar diameter 2 delignified	2.493245	1.27E-02	1.00E+00
	Poplar diameter 2 untreated	2.156271	3.11E-02	1.00E+00
	Spruce diameter 1 delignified	-1.71742	8.59E-02	1.00E+00
	Spruce diameter 1 untreated	-2.76654	5.67E-03	8.27E-01
	Spruce diameter 2 delignified	2.219164	2.65E-02	1.00E+00
	Spruce diameter 2 untreated	-0.96533	3.34E-01	1.00E+00
Beech diameter 1 untreated	Beech diameter 2 delignified	-0.01603	9.87E-01	9.87E-01
	Beech diameter 2 untreated	1.151327	2.50E-01	1.00E+00
	Douglas fir diameter 1 delignified	-4.82911	1.37E-06	2.85E-04
	Douglas fir diameter 1 untreated	-4.47363	7.69E-06	1.51E-03
	Douglas fir diameter 2 delignified	-0.33657	7.36E-01	1.00E+00
	Douglas fir diameter 2 untreated	0.462556	6.44E-01	1.00E+00
	Poplar diameter 1 delignified	-1.24757	2.12E-01	1.00E+00
	Poplar diameter 1 untreated	-1.7827	7.46E-02	1.00E+00
	Poplar diameter 2 delignified	0.705654	4.80E-01	1.00E+00

	Poplar diameter 2 untreated	0.675265	5.00E-01	1.00E+00
Beech diameter 1 untreated	Spruce diameter 1 delignified	-3.0078	2.63E-03	4.13E-01
	Spruce diameter 1 untreated	-3.81862	1.34E-04	2.40E-02
	Spruce diameter 2 delignified	0.463939	6.43E-01	1.00E+00
	Spruce diameter 2 untreated	-2.17435	2.97E-02	1.00E+00
Beech diameter 2 delignified	Beech diameter 2 untreated	1.277242	2.02E-01	1.00E+00
	Douglas fir diameter 1 delignified	-5.38119	7.40E-08	1.64E-05
	Douglas fir diameter 1 untreated	-4.88459	1.04E-06	2.19E-04
	Douglas fir diameter 2 delignified	-0.35838	7.20E-01	1.00E+00
	Douglas fir diameter 2 untreated	0.522732	6.01E-01	1.00E+00
	Poplar diameter 1 delignified	-1.39545	1.63E-01	1.00E+00
	Poplar diameter 1 untreated	-1.93682	5.28E-02	1.00E+00
	Poplar diameter 2 delignified	0.819293	4.13E-01	1.00E+00
	Poplar diameter 2 untreated	0.755743	4.50E-01	1.00E+00
	Spruce diameter 1 delignified	-3.39137	6.95E-04	1.18E-01
	Spruce diameter 1 untreated	-4.16706	3.09E-05	5.80E-03
	Spruce diameter 2 delignified	0.545213	5.86E-01	1.00E+00
	Spruce diameter 2 untreated	-2.36586	1.80E-02	1.00E+00
Beech diameter 2 untreated	Douglas fir diameter 1 delignified	-6.09033	1.13E-09	2.58E-07
	Douglas fir diameter 1 untreated	-5.62496	1.86E-08	4.17E-06
	Douglas fir diameter 2 delignified	-1.59779	1.10E-01	1.00E+00
	Douglas fir diameter 2 untreated	-0.68877	4.91E-01	1.00E+00
	Poplar diameter 1 delignified	-2.577	9.97E-03	1.00E+00

	Poplar diameter 1 untreated	-2.93403	3.35E-03	5.15E-01
Beech diameter 2 untreated	Poplar diameter 2 delignified	-0.62378	5.33E-01	1.00E+00
	Poplar diameter 2 untreated	-0.47606	6.34E-01	1.00E+00
	Spruce diameter 1 delignified	-4.33724	1.44E-05	2.78E-03
	Spruce diameter 1 untreated	-4.96995	6.70E-07	1.43E-04
	Spruce diameter 2 delignified	-0.8655	3.87E-01	1.00E+00
	Spruce diameter 2 untreated	-3.32568	8.82E-04	1.48E-01
Douglas fir diameter 1 delignified	Douglas fir diameter 1 untreated	-0.07151	9.43E-01	1.00E+00
	Douglas fir diameter 2 delignified	5.022812	5.09E-07	1.10E-04
	Douglas fir diameter 2 untreated	5.335815	9.51E-08	2.10E-05
	Poplar diameter 1 delignified	4.357284	1.32E-05	2.55E-03
	Poplar diameter 1 untreated	2.876261	4.02E-03	6.08E-01
	Poplar diameter 2 delignified	6.572028	4.96E-11	1.15E-08
	Poplar diameter 2 untreated	5.568826	2.56E-08	5.74E-06
	Spruce diameter 1 delignified	2.361362	1.82E-02	1.00E+00
	Spruce diameter 1 untreated	0.646018	5.18E-01	1.00E+00
	Spruce diameter 2 delignified	6.297947	3.02E-10	6.94E-08
	Spruce diameter 2 untreated	2.447226	1.44E-02	1.00E+00
Douglas fir diameter 1 untreated	Douglas fir diameter 2 delignified	4.564045	5.02E-06	1.03E-03
	Douglas fir diameter 2 untreated	4.936186	7.97E-07	1.70E-04
	Poplar diameter 1 delignified	3.918137	8.92E-05	1.63E-02
	Poplar diameter 1 untreated	2.690931	7.13E-03	1.00E+00
	Poplar diameter 2 delignified	5.871357	4.32E-09	9.86E-07

	Poplar diameter 2 untreated	5.148895	2.62E-07	5.71E-05
Douglas fir diameter 1 untreated	Spruce diameter 1 delignified	2.157899	3.09E-02	1.00E+00
	Spruce diameter 1 untreated	0.655007	5.12E-01	1.00E+00
	Spruce diameter 2 delignified	5.629641	1.81E-08	4.08E-06
	Spruce diameter 2 untreated	2.299277	2.15E-02	1.00E+00
Douglas fir diameter 2 delignified	Douglas fir diameter 2 untreated	0.843276	3.99E-01	1.00E+00
	Poplar diameter 1 delignified	-1.01233	3.11E-01	1.00E+00
	Poplar diameter 1 untreated	-1.61628	1.06E-01	1.00E+00
	Poplar diameter 2 delignified	1.202416	2.29E-01	1.00E+00
	Poplar diameter 2 untreated	1.076286	2.82E-01	1.00E+00
	Spruce diameter 1 delignified	-3.00825	2.63E-03	4.15E-01
	Spruce diameter 1 untreated	-3.84652	1.20E-04	2.16E-02
	Spruce diameter 2 delignified	0.928336	3.53E-01	1.00E+00
	Spruce diameter 2 untreated	-2.04531	4.08E-02	1.00E+00
Douglas fir diameter 2 untreated	Poplar diameter 1 delignified	-1.78168	7.48E-02	1.00E+00
	Poplar diameter 1 untreated	-2.24526	2.48E-02	1.00E+00
	Poplar diameter 2 delignified	0.17154	8.64E-01	1.00E+00
	Poplar diameter 2 untreated	0.212708	8.32E-01	1.00E+00
	Spruce diameter 1 delignified	-3.54192	3.97E-04	6.83E-02
	Spruce diameter 1 untreated	-4.28118	1.86E-05	3.55E-03
	Spruce diameter 2 delignified	-0.07018	9.44E-01	1.00E+00
	Spruce diameter 2 untreated	-2.63691	8.37E-03	1.00E+00
Poplar diameter 1 delignified	Poplar diameter 1 untreated	-0.81092	4.17E-01	1.00E+00

	Poplar diameter 2 delignified	2.392197	1.67E-02	1.00E+00
Poplar diameter 1 delignified	Poplar diameter 2 untreated	2.027295	4.26E-02	1.00E+00
	Spruce diameter 1 delignified	-2.15584	3.11E-02	1.00E+00
	Spruce diameter 1 untreated	-3.1618	1.57E-03	2.52E-01
	Spruce diameter 2 delignified	2.096156	3.61E-02	1.00E+00
	Spruce diameter 2 untreated	-1.26316	2.07E-01	1.00E+00
Poplar diameter 1 untreated	Poplar diameter 2 delignified	2.764138	5.71E-03	8.28E-01
	Poplar diameter 2 untreated	2.457964	1.40E-02	1.00E+00
	Spruce diameter 1 delignified	-0.94932	3.42E-01	1.00E+00
	Spruce diameter 1 untreated	-2.03592	4.18E-02	1.00E+00
	Spruce diameter 2 delignified	2.522422	1.17E-02	1.00E+00
	Spruce diameter 2 untreated	-0.39165	6.95E-01	1.00E+00
Poplar diameter 2 delignified	Poplar diameter 2 untreated	0.074074	9.41E-01	1.00E+00
	Spruce diameter 1 delignified	-4.54804	5.41E-06	1.10E-03
	Spruce diameter 1 untreated	-5.11502	3.14E-07	6.81E-05
	Spruce diameter 2 delignified	-0.29604	7.67E-01	1.00E+00
	Spruce diameter 2 untreated	-3.21638	1.30E-03	2.12E-01
Poplar diameter 2 untreated	Spruce diameter 1 delignified	-3.78753	1.52E-04	2.69E-02
	Spruce diameter 1 untreated	-4.49389	6.99E-06	1.39E-03
	Spruce diameter 2 delignified	-0.31579	7.52E-01	1.00E+00
	Spruce diameter 2 untreated	-2.84962	4.38E-03	6.57E-01
Spruce diameter 1 delignified	Spruce diameter 1 untreated	-1.40156	1.61E-01	1.00E+00
	Spruce diameter 2 delignified	4.251998	2.12E-05	4.03E-03

	Spruce diameter 2 untreated	0.497077	6.19E-01	1.00E+00
Spruce diameter 1 untreated	Spruce diameter 2 delignified	4.873304	1.10E-06	2.30E-04
	Spruce diameter 2 untreated	1.64427	1.00E-01	1.00E+00
Spruce diameter 2 delignified	Spruce diameter 2 untreated	-2.97467	2.93E-03	4.58E-01

Table S2: Statistical significances of tracheid wall thickness. Kruskal–Wallis ANOVA (p = 6.09282E-07) with Dunn's post-hoc test and Holm's correction.

Group 1	Group 2	Z	P.unadj	P.adj
Balsa delignified	Balsa untreated	3.6949	2.20E-04	9.24E-03
	Beech delignified	0.7877	4.31E-01	1.00E+00
	Beech untreated	1.7843	7.44E-02	1.00E+00
	Douglas fir delignified	3.4927	4.78E-04	1.91E-02
	Douglas fir untreated	0.9661	3.34E-01	1.00E+00
	Poplar delignified	0.4976	6.19E-01	1.00E+00
	Poplar untreated	1.0097	3.13E-01	1.00E+00
	Spruce delignified	-0.2187	8.27E-01	1.00E+00
	Spruce untreated	-2.7592	5.79E-03	1.85E-01
Balsa untreated	Beech delignified	-2.7228	6.47E-03	2.01E-01
	Beech untreated	-1.5776	1.15E-01	1.00E+00
	Douglas fir delignified	-0.7477	4.55E-01	1.00E+00
	Douglas fir untreated	-2.2532	2.42E-02	6.79E-01
	Poplar delignified	-3.1453	1.66E-03	6.30E-02
	Poplar untreated	-2.2172	2.66E-02	7.18E-01
	Spruce delignified	-3.6889	2.25E-04	9.23E-03
	Spruce untreated	-5.3293	9.86E-08	4.44E-06
Beech delignified	Beech untreated	0.9946	3.20E-01	1.00E+00
	Douglas fir delignified	2.3167	2.05E-02	5.95E-01
	Douglas fir untreated	0.2546	7.99E-01	1.00E+00
	Poplar delignified	-0.3121	7.55E-01	1.00E+00
	Poplar untreated	0.2939	7.69E-01	1.00E+00
	Spruce delignified	-0.9285	3.53E-01	1.00E+00
	Spruce untreated	-3.1151	1.84E-03	6.80E-02
Beech untreated	Douglas fir delignified	1.0321	3.02E-01	1.00E+00
	Douglas fir untreated	-0.6756	4.99E-01	1.00E+00
	Poplar delignified	-1.3237	1.86E-01	1.00E+00
	Poplar untreated	-0.6397	5.22E-01	1.00E+00
	Spruce delignified	-1.8673	6.19E-02	1.00E+00
	Spruce untreated	-3.7517	1.76E-04	7.55E-03
Douglas fir delignified	Douglas fir untreated	-1.7942	7.28E-02	1.00E+00

	Poplar delignified	-2.8161	4.86E-03	1.60E-01
	Poplar untreated	-1.7537	7.95E-02	1.00E+00
Douglas fir delignified	Spruce delignified	-3.4593	5.42E-04	2.11E-02
	Spruce untreated	-5.2645	1.41E-07	6.18E-06
Douglas fir untreated	Poplar delignified	-0.5436	5.87E-01	1.00E+00
	Poplar untreated	0.0359	9.71E-01	9.71E-01
	Spruce delignified	-1.0872	2.77E-01	1.00E+00
	Spruce untreated	-3.0761	2.10E-03	7.34E-02
Poplar delignified	Poplar untreated	0.5851	5.58E-01	1.00E+00
	Spruce delignified	-0.6658	5.06E-01	1.00E+00
	Spruce untreated	-3.0084	2.63E-03	8.93E-02
Poplar untreated	Spruce delignified	-1.1287	2.59E-01	1.00E+00
	Spruce untreated	-3.1120	1.86E-03	6.69E-02
Spruce delignified	Spruce untreated	-2.4648	1.37E-02	4.11E-01

Table S3: Shrinking/Expansion rate of the diameters after delignification. Median values of the diameters of untreated and delignified samples with the shrinking rate (-) and the expansion rate (+) after delignification.

**Species**	**Treatment**	**Shape**	**Diameter [µm]**		**Shrinkage (-) /Expansion (+)**
			**untreated**	**delignified**	
spruce (*P. abies*)	Diameter1	rectangular	40.3	26.9	-33.3%
	Diameter 2		26.7	9.9	-63%
beech (*F. sylvatica*)	Diameter1	rectangular	17.2	21.8	+26.5%
	Diameter 2		10.8	16.7	+53.5%
balsa (*O. pyramidale*)	Diameter1	hexagonal	32.8	26.6	-18.9%
	Diameter1	rectangular	37.7	29.4	-22.1%
	Diameter 2		24.7	22.4	-9.4%
Douglas fir (*P. menziesii*)	Diameter1	oval	43	40.9	-4.8%
	Diameter 2	oval	12.7	17.6	+38.4%
poplar (*P. spp.*)	Diameter1	pentagonal	23.2	20.6	-11%
	Diameter 2		14.2	14.5	+1.7%

Table S4: Shrinking/Expansion rate of the wall thickness after delignification. Median values of the wall thickness of untreated and delignified samples with the shrinking rate (-) and the expansion rate (+) after delignification.

Species	Treatment	Wall thickness [µm]	Shrinkage (-) /Expansion (+)
spruce *(P. abies)*	untreated	4.1	-58.4%
	delignified	1.7	
beech *(F. sylvatica)*	untreated	1.3	+11.5%
	delignified	1.4	
balsa *(O. pyramidale)*	untreated	0.8	+165.8%
	delignified	2.1	
Douglas fir *(P. menziesii)*	untreated	1.4	-40.9%
	delignified	0.8	
poplar *(P.* spp.*)*	untreated	1.5	+9.2%
	delignified	1.6	
